# Abdominal Adiposity Distribution in Diabetic/Prediabetic and Nondiabetic Populations: A Meta-Analysis

**DOI:** 10.1155/2014/697264

**Published:** 2014-11-26

**Authors:** Jane J. Lee, S. Natasha Beretvas, Jeanne H. Freeland-Graves

**Affiliations:** ^1^Department of Nutritional Sciences, The University of Texas at Austin, 1 University Station A2703, Austin, TX 78712, USA; ^2^Department of Educational Psychology, The University of Texas at Austin, 1 University Station D5800, Austin, TX 78712, USA

## Abstract

Excess fat in the abdomen can be classified generally as visceral and subcutaneous adiposity. Evidence suggests that visceral adiposity has greater implications for diabetes than other fat depots. The purpose of this study is to explore the disparities in the distribution of abdominal adiposity in diabetic/prediabetic and nondiabetic populations and to identify moderators that influence the pattern of central obesity via a meta-analysis technique. The Hedges' *g* was used as a measure of effect size and 95% confidence interval was computed. A total of 41 relevant studies with 101 effect sizes were retrieved. Pooled effect sizes for visceral and subcutaneous adiposity were 0.69 and 0.42, respectively. Diabetic/prediabetic populations exhibited greater visceral and subcutaneous adiposity compared to nondiabetic populations (*Z* = 10.35, *P* < 0.05). Significant moderator effects of gender (*Z* = −2.90) and assessment method of abdominal adiposity (*Z* = −2.17) were found for visceral fat (*P* < 0.05), but not for subcutaneous fat. Type of health condition influenced both visceral (*Z* = −5.10) and subcutaneous (*Z* = −7.09) abdominal adiposity volumes (*P* < 0.05). Abdominal adiposity distributions were significantly altered in the diabetic/prediabetic population compared to the nondiabetic population. Gender, assessment method of abdominal adiposity, and type of health conditions (diabetic/prediabetics) were identified as crucial moderators that influence the degree of abdominal adiposity.

## 1. Introduction

Central obesity is a significant health problem associated with glucose intolerance, insulin resistance, metabolic perturbations, hyperinsulinemia, and progression to type 2 diabetes mellitus [1, 2]. According to the World Health Organization, 10.1% of the U.S. population over 25 years old have raised fasting blood glucose (≥7.0 mmol/L versus 5.5 mmol/L for normal) [3]. In adults over 20 years and 65 years old, the incidence of diabetes was 11.3% and 26.9%, respectively [4]. Diabetes accounted for 3.4% of all deaths in the United States in 2011 [5] and 4.8 million deaths worldwide in 2012 [6]. By 2030, this global disease is expected to be the seventh most common cause of death [7, 8]. Thus, the health consequences of diabetes are severe and approaching epidemic proportions. The risks of being diagnosed with diabetes are critical, as it is linked to heart disease, blindness, kidney failure, retinopathy, limb amputation, and other physical ailments [9, 10]. Obesity is one of the most crucial contributors that increase the risk of being diagnosed with diabetes [11].

Abdominal (central) obesity is of concern as it has been shown to have a greater association with diabetes or metabolic syndrome, as opposed to overall obesity [2, 12–14]. Generally, excess fat in the abdomen is classified as visceral adiposity (abdominal fat depots around organs), subcutaneous adiposity (abdominal fat depots underneath skin), and ectopic fat (fat depots in locations not associated with accumulation of adipose tissue) [15]. Among these fat depots, more evidence suggests that visceral adiposity has a more significant impact on diabetes-related risk factors, than that found in subcutaneous depots [2, 12, 16, 17]. To date, no study has examined the consistency of the different types of the abdominal adiposity distribution (visceral and subcutaneous) between diabetic/prediabetic and nondiabetic populations. Thus, a meta-analysis was conducted to explore possible disparities of the abdominal adiposity distribution between these two groups.

Women are known to have a higher percentage of fat than men, even after adjustment for body mass index (BMI) and age [18, 19]. In terms of central obesity, men are more prone to android adiposity (apple-shaped) with greater abdominal adiposity, as compared to women who are more likely to exhibit gynoid adiposity (pear-shaped) [20]. However, these gender differences in body shape may not be related directly to the degree of visceral and subcutaneous adiposity. Westerbacka et al. (2004) reported that women exhibited twice as much subcutaneous adiposity as men; but, gender did not appear to influence visceral adipose tissue [21]. However, in obese men and women with matched age and BMI, women had a higher total and subcutaneous adiposity in the abdominal area, whereas men had a greater visceral adiposity [22]. The present meta-analysis explored gender as a moderator that influences the degree of central obesity.

Traditional anthropometric measurements used to assess risk for diabetes [23] that indicate central obesity include waist circumference, sagittal abdominal diameter, waist-to-hip ratio, waist-to-height ratio, and skinfold thicknesses [23–25]. Of these, waist circumference and sagittal abdominal diameter are the most reflective indicators of diabetes risk [26, 27]. Waist-to-height ratio also has been demonstrated to be a better predictor of diabetes than other anthropometric variables, including waist circumference [28]. Yet, these traditional anthropometric parameters remain imprecise due to lack of differentiation between visceral and subcutaneous depots. At present, computed tomography (CT) and magnetic resonance imaging (MRI) are the advanced instruments of choice to quantify central obesity, as both identify the volumes of visceral and subcutaneous adipose tissue [29–31]. In this research, the moderating effect of CT versus MRI method on abdominal adiposity distribution was compared in diabetic/prediabetic versus nondiabetic populations.

Prediabetes is a continuum between normal health and diabetes that is characterized by increased blood glucose and fasting plasma glucose, as well as impaired glucose tolerance [10, 32]. Between 2005 and 2008, 35% and 50% of adults in the United States over 20 years and 65 years old were diagnosed with prediabetes, respectively [4]. With progression to type 2 diabetes, fasting blood glucose and glucose tolerance response become even higher [10]. This research explored the degree of the central obesity distribution within diabetic and prediabetic groups and conducted comparisons to the nondiabetic group.

The aims of this study were (1) to explore the degree and distribution of abdominal adipose tissue volumes (visceral and subcutaneous) in diabetic/prediabetic and nondiabetic groups and (2) to identify the influence of moderators (gender, method of assessment, and type of health condition (diabetes/prediabetes)).

## 2. Materials and Methods

### 2.1. Study Selection

**Figure 1:**
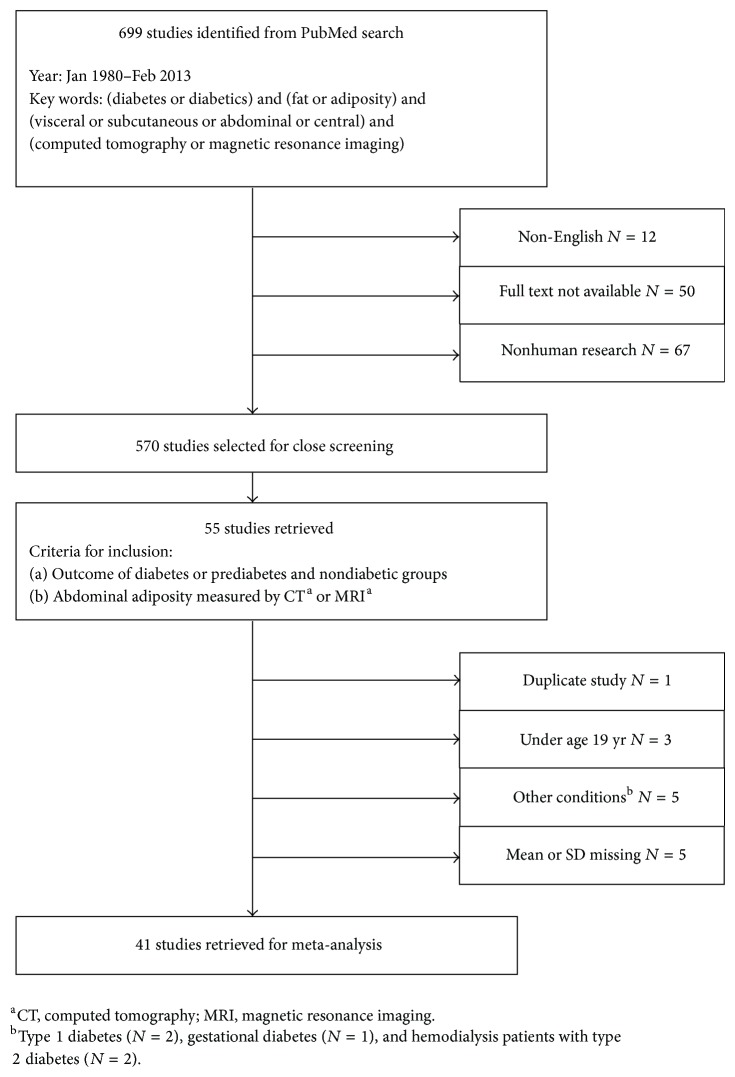
Flow diagram illustrating process of study selection for meta-analysis with inclusion and exclusion criteria.

A systematic meta-analysis was performed by searching relevant publications via PubMed databases (http://www.ncbi.nlm.nih.gov/pubmed) from January 1980 to February 2013. A total of 699 papers were identified via the keywords “diabetes or diabetics,” “fat or adiposity,” “visceral or subcutaneous or abdominal or central,” and “computed tomography or magnetic resonance imaging (Figure 1).”

Figure 1 is a flow diagram that illustrates the process of study selection for the meta-analysis with inclusion and exclusion criteria. Among the identified publications, 129 articles were excluded that were not published in English, did not have full text available, or were not based on human research. Inclusion criteria were studies that compared the outcomes between diabetic/prediabetic and nondiabetic groups in which the diabetic/prediabetic groups were subjects who were diabetics or exhibited prediabetic conditions, such as insulin resistance, insulin sensitivity, impaired glucose tolerance, or metabolic syndrome; and the nondiabetic groups involve participants who were free of diabetic diseases. Studies were included only if the outcomes of visceral and subcutaneous adiposity volumes were measured by either CT or MRI. In order to reduce the heterogeneity, the 55 retrieved studies were then subject to exclusion criteria of using duplicate data, being subjects under 19 years, or being diagnosed with other types of diabetes (type 1 diabetes mellitus, gestational diabetes) or on hemodialysis. Research that did not report group means and/or standard deviations of abdominal adiposity volumes also was eliminated in order to obtain valid effect sizes for the meta-analysis.

### 2.2. Data Extraction

Each study that was selected from the screening process contained one or more effect sizes, as this research explored two different types of abdominal adiposity (visceral and subcutaneous) within the same population. Therefore, the data extraction was performed based on a multiple-outcome study setting [74].

The mean value and the standard deviation of visceral and subcutaneous adiposity volumes and sample sizes of the diabetic/prediabetic and nondiabetic groups from each experiment were obtained to calculate the outcome-specific effect size estimate (Hedges' *g* or standardized mean differences). Standard errors of the mean were obtained and converted into standard deviations in studies that reported only the standard errors of the mean instead of the standard deviations. In addition, the proportion of men in the subject sample, method of assessment to measure abdominal adipose tissue, and information on participants' characteristics related to diabetes were collected in order to conduct moderator analyses.

## 3. Statistical Analysis

### 3.1. Estimation of Effect Sizes

Pooled sample variances were calculated using degrees of freedom and standard deviations of diabetic/prediabetic and nondiabetic groups. Computation of the pooled sample variance involved weighting the estimated sample variance with its sample size. Consequently, more weight was given to the variance with a larger sample size by using the pooled sample variance.

The effect size estimate for each study for visceral and subcutaneous adiposity was computed by dividing the differences between the mean values of the abdominal adiposity volumes of the control and treatment groups by the square root of the pooled sample variance. This method incorporated Hedges' *g*, which is the standardized mean difference [75]. The adiposity volumes of the diabetic/prediabetic group were hypothesized to be greater than the nondiabetic group. Hence, the mean value of the abdominal adiposity volumes of the nondiabetic group was subtracted from the mean value of the abdominal adiposity volumes of the diabetics/prediabetics to avoid negative effect size values. Hedges bias-correction for *g* was used to obtain the unbiased estimator of the effect size [74]. A 95% confidence interval for population effect size for each study was obtained.

### 3.2. Generalized Least Squares Method and Pooled Effect Sizes

One or more effect sizes were extracted from the same study because this research involves a meta-analysis of exploring the multivariate outcomes, visceral and subcutaneous adiposity. The estimated pooled average effect size was acquired by weighting each effect size estimate by the inverse of its variance, as well as the covariance between pairs of effect size estimates. This weighting process was conducted by using generalized least squares (GLS) [76].

The independency of the visceral and subcutaneous adiposity effect sizes extracted from the same study could not be assumed in the multivariate study setting. Effect sizes from the same study were measured by utilizing identical diabetic/prediabetic and nondiabetic groups and incorporating adiposity measures that are related. The covariance between estimated effect sizes of visceral and subcutaneous adiposity was acquired in order to model the dependency between effect sizes of the two different abdominal adiposity volumes, visceral and subcutaneous fats. Estimates of the correlation between visceral and subcutaneous adiposity volumes were collected from the included studies in the meta-analysis screening procedure.

### 3.3. *Q*-Statistics and Homogeneity of Effects

The significance of the effects of visceral and subcutaneous adiposity was evaluated by computing *Q*_*B*_ [74]. This omnibus test was conducted to assess whether the two groups, diabetic/prediabetic and nondiabetic, differed in the distribution of abdominal adiposity. Rejecting the *Q*_*B*_ statistic implied that at least one of the group differences in abdominal adiposity distribution was statistically significant.

The homogeneity of effects was performed by *Q*_*WB*_ to examine if the quantity of visceral and subcutaneous adiposity volumes was consistent across the studies [74]. The null hypothesis was that the degrees of abdominal adiposity volumes are homogeneous between the studies. The alternative hypothesis was that the abdominal adiposity volumes were not the same among the studies.

The significance of the *Q*_*WB*_ statistic was determined by comparing the *Q*_*WB*_ statistic to the *X* ^2^ distribution, with relevant degrees of freedom. The degrees of freedom were acquired by subtracting the number of effect parameters (visceral and subcutaneous adiposity) from the number of effect sizes retrieved from the screening process. Rejecting the *Q*_*WB*_ statistic indicates that the amount of visceral and subcutaneous adiposity volumes significantly differ among groups. An alpha level of 0.05 was used for each statistical test.

### 3.4. Individual Effects of Visceral and Subcutaneous Adiposity

The individual significance level was determined when group differences in abdominal adiposity were statistically significant, which was supported by *Q*_*B*_. Three contrast tests independently examined the significance of visceral and subcutaneous adiposity volumes and assessed the significant differences between visceral and subcutaneous adiposity. The cutoff point for the two-tailed test with 0.05 alpha level was ±2.13, based on the assumption that *Z*-statistics follows the normal distribution. The values of *Z*-statistics larger than 2.13 or smaller than −2.13 were regarded to be statistically significant at an alpha level of 0.05 by applying the Bonferroni adjustment with three pairwise comparisons [77].

### 3.5. Moderator Effects on Visceral and Subcutaneous Adiposity

Two individual statistical tests were conducted in order to evaluate the gender moderator effect (men and women) in visceral and subcutaneous adiposity volumes among diabetic/prediabetic and nondiabetic groups. A contrast test also was conducted for differences in gender effects on visceral and subcutaneous adiposity effects. To identify the adiposity type-specific gender effect, *Z*-statistics was obtained for visceral and subcutaneous adiposity volumes effects. Studies that did not report the information of the gender of the participants were excluded from the gender moderator effect analysis.

Two individual statistical tests were conducted to investigate the method of assessment (CT and MRI) on visceral and subcutaneous adiposity volumes. A contrast test was conducted via *Z*-test to explore the differences in the method of assessment effect on visceral and subcutaneous adiposity volumes between diabetic/prediabetic and nondiabetic populations.

Finally, two individual statistical tests were executed to explore the type of health conditions of the diabetic/prediabetic group moderator effect on visceral and subcutaneous adiposity. A contrast test was conducted to identify the differences in type of health conditions of diabetic/prediabetic group moderator effect on visceral and subcutaneous adiposity. To further investigate the adiposity type-specific moderator effect of type of health conditions of treatment group, *Z*-statistics was acquired for the visceral and subcutaneous adiposity volume effects. The cutoff value for the individual tests was ±2.13, which was followed by Bonferroni adjustment with three pairwise comparisons [77].

Evidence of publication bias was analyzed by a funnel plot, which is a visual summary of a meta-analysis dataset [78]. Scatter plots of the effect size measure (bias corrected hedge's *g* or standardized mean differences) against standard error of the estimated effect size were created and evaluated by the author. Data without publication bias is expected to generate a funnel shaped scatter plot that follows the distribution of the effect sizes. Greater variabilities are expected in studies with smaller sample sizes, as opposed to the studies with larger sample sizes when publication bias is absent.

All statistical analyses were performed using SAS (version 9.3) PROC IML [79]. The equations and methods that were incorporated in the analyses were obtained from The* Handbook of Research Synthesis and Meta-Analysis* [74].

## 4. Results

### 4.1. Study Selection

A total of 101 effect sizes were extracted from 41 studies, 54 effect sizes for visceral adiposity, and 47 effect sizes for subcutaneous adiposity. Tables 1 and 2 summarize the characteristics and the information of moderators of the included studies. Among the 41 studies selected for the meta-analysis, 22,552 subjects were included. In particular, 12,078 participants (3,227 for diabetic/prediabetic groups and 8,851 for nondiabetic groups) were involved in visceral adiposity effect size measurement and 10,474 (2,827 for diabetic/prediabetic groups and 7,647 for nondiabetic groups) individuals were incorporated for subcutaneous adiposity effect size assessment.

The mean age of the participants ranged from 28 to 74 years. The mean value of BMI ranged from 21.0 to 41.9, encompassing normal to morbidly obese weight status. Participants included men or women or both genders, as well as Caucasians, Hispanics, African-Americans, Native American, and Asians. Among 54 visceral adiposity effect sizes, 38 effect sizes were measured via CT and 16 effect sizes were assessed by MRI. Similarly, throughout 47 subcutaneous effect sizes, 34 effect sizes were quantified via CT and 13 effect sizes were acquired by MRI (Tables 1 and 2).

The physical conditions of the diabetic/prediabetic groups were divided into two categories, type 2 diabetes mellitus or prediabetic groups. Type 2 diabetic group consisted of patients with diabetes without any other known clinical diseases. Prediabetic groups included nondiabetic subjects with metabolic syndrome, insulin resistance, metabolic syndrome, metabolically obese normal weight individuals with normal glucose tolerance, impaired glucose tolerance, impaired fasting glucose, insulin sensitivity, impaired glucose tolerance, and altered insulin-glucose homeostasis (Tables 1 and 2).

### 4.2. Population and Pooled Effect Sizes

**Figure 2:**
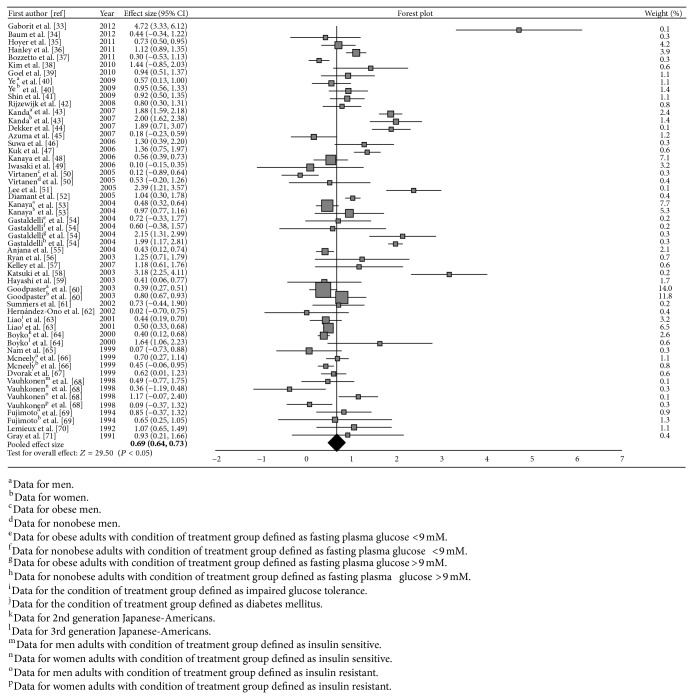
Forest plot of weighted population and pooled effect size estimates and its 95% confidence intervals representing disparities in visceral adiposity distribution among diabetic/prediabetic versus nondiabetic groups.

**Figure 3:**
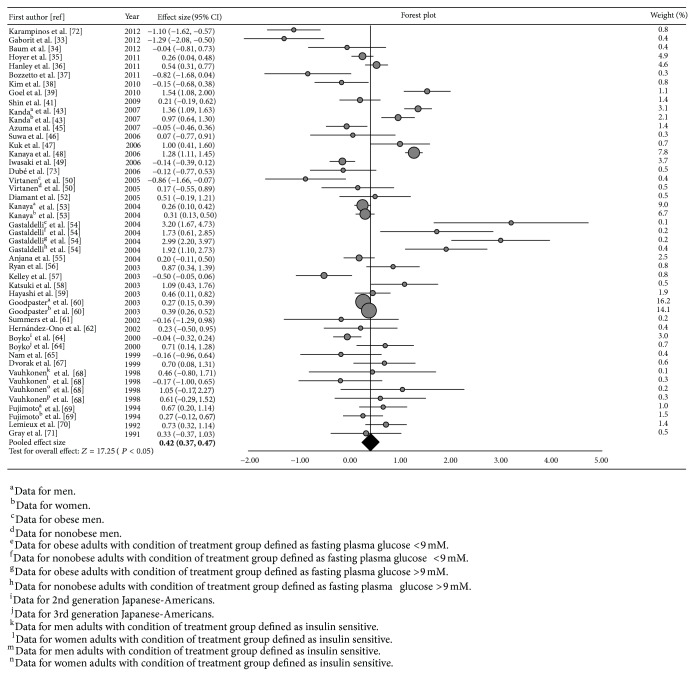
Forest plot of weighted population and pooled effect size estimates and its 95% confidence intervals representing disparities in subcutaneous adiposity distribution among diabetic/prediabetic versus nondiabetic groups.

Final multivariate pooled effect size estimates of visceral and subcutaneous adiposity by using GLS method were 0.69 and 0.42, respectively. The 95% confidence interval of the pooled effect size was (0.64, 0.73) for visceral adiposity and (0.37, 0.47) for subcutaneous adiposity (Figures 2 and 3). These are illustrated in the forest plots constructed by weighted population effect sizes. Each square symbol in Figure 2 and each circle symbol in Figure 3 indicate the weighted population effect size for each study. The lines that are extended from the symbols contain the limits of the 95% confidence interval. The sizes of the square and circle symbols reflect the degree of weight of its population effect size. The diamond symbol at the bottom of the last row in each table illustrates the weighted pooled effect size, which was acquired by weighting the population effect sizes. The black line intersecting the diamond symbol demonstrates a 95% confidence interval for the weighted pooled effect size. Due to the large sample sizes involved in this study, the 95% confidence interval for the weighted pooled effect size is so narrow that it is indecipherable.

### 4.3. *Q*-Statistics and Homogeneity of Effects

The value of *Q*_*B*_ computed was 900.07, which was statistically significant (*P* < 0.05). Results of this test indicated that at least one of the abdominal adiposity effect sizes is significantly different from zero.

The value of *Q*_*WB*_ was 731.70 (*P* < 0.05), and this test was derived to evaluate the homogeneity of the effect sizes among visceral and subcutaneous adiposity volumes in diabetic/prediabetic and nondiabetic groups. Thus, evidence was found for heterogeneity in the effect sizes of visceral and subcutaneous adiposity between diabetic/prediabetic and nondiabetic groups.

### 4.4. Individual Effects of Visceral and Subcutaneous Adiposity

As a consequence of the rejection of *Q*_*B*_ and *Q*_*WB*_ statistics, the individual visceral and subcutaneous adiposity effects were tested for statistical significance. The results of *Z*-tests for visceral and subcutaneous effect sizes were 29.50 and 17.25, respectively. Both *Z*-statistic values were statistically significant (*P* < 0.05, *Z* > 2.13) indicating that visceral and subcutaneous adiposity volumes differed significantly between diabetic/prediabetic versus nondiabetic populations. The diabetic/prediabetic groups exhibited a higher amount of visceral and subcutaneous adiposity, as opposed to individuals without a diagnosis of diabetes mellitus.

The contrast test for the visceral and subcutaneous adiposity volumes in diabetic/prediabetic versus nondiabetic populations also was statistically different (*Z* = 10.35, *P* < 0.05). The direction of the *Z*-test was positive (*Z* > 2.13), implying that the disparities between diabetic/prediabetic and nondiabetic populations were significantly greater for visceral adiposity, as opposed to that for subcutaneous adiposity.

### 4.5. Moderator Effects on Visceral and Subcutaneous Adiposity

#### 4.5.1. Gender Effect

Final multivariate pooled estimates by using GLS were 0.77 for the proportion of men and women for visceral adiposity and 0.40 for proportion of men and women for subcutaneous adiposity.

The *Q*_*B*_ value for testing if at least one of the four elements (proportion of men and women for visceral and subcutaneous adiposity) differs from zero was 862.87 (*P* < 0.05). This result suggests that at least one of the four effects is different from zero.

*Z*-Statistics for independently testing the gender as a moderator effect on visceral and subcutaneous adiposity was −2.90 and 0.36, respectively. The *Z*-statistics for gender effect on the visceral adiposity effect size was significantly negative (*P* < 0.05, *Z* < −2.13), whereas the effect of gender on the subcutaneous effect size was positive but not statistically significant (*P* > 0.05, −2.13 < *Z* < 2.13). These results denoted that the differences in visceral adiposity volumes between diabetic/prediabetic versus nondiabetic groups were significantly smaller for men, as opposed to women. No significant gender effect was observed for differences in abdominal adiposity volume between diabetic/prediabetic and nondiabetic groups for subcutaneous adiposity.

The *Z*-statistics for the contrast of gender as a moderator effect was −3.05, which was significantly negative (*P* > 0.05, *Z* < −2.13).

#### 4.5.2. Method of Assessment (CT or MRI) Effect

Final multivariate pooled estimates by using GLS were 0.67 for CT in visceral fat, 0.84 for MRI in visceral fat, 0.42 for CT in subcutaneous fat, and 0.42 for MRI in subcutaneous fat.

The *Q*_*B*_ value for testing, if at least one of the four elements (CT and MRI in visceral and subcutaneous adiposity) differs from zero, yielded significant results of 904.87 (*P* < 0.05). This finding suggests that significant instrument moderator effect exists on the abdominal adiposity effect sizes among diabetic/prediabetic and nondiabetic populations.

The *Z*-statistics for independently testing the instrument as a moderator effect on visceral and subcutaneous adiposity effect sizes was −2.17 and −0.06, respectively. *Z*-Statistics for visceral adiposity was negatively significant (*P* < 0.05, *Z* < −2.13), whereas subcutaneous adiposity effect size for testing the method of assessment effect was not significant (*P* > 0.05, −2.13 < *Z* < 2.13). Thus, the differences in visceral adiposity between diabetic/prediabetic versus nondiabetic groups were smaller when CT was used to measure abdominal adiposity. The method of assessment effect was not observed for detecting differences in subcutaneous adiposity distribution.

The contrast test for distinguishing the type of instrument as a moderator on abdominal adiposity volumes was −1.48, which was negative but not significant (*P* < 0.05, *Z* < −2.13).

#### 4.5.3. Type of Health Conditions Effect (Diabetic or Prediabetic)

Final multivariate pooled estimates by using GLS were 0.76 for type 2 diabetic participants in visceral adiposity, 0.89 for prediabetic group in visceral adiposity, 0.33 for diabetic individuals in subcutaneous group, and 0.62 for prediabetic group in subcutaneous group.

The *Q*_*B*_ value for testing, if at least one of the four elements (diabetic and prediabetic groups in visceral and subcutaneous adiposity) differs from zero, was 957.23 (*P* < 0.05). This demonstrates that significant types of health conditions for diabetic/prediabetic group moderator effect exist on the differences in abdominal adiposity distribution between diabetic and prediabetic groups.

*Z*-Statistics for testing type of health conditions as a moderator effect on abdominal adiposity was −5.10 for visceral adiposity and −7.09 for subcutaneous adiposity (*P* < 0.05, *Z* < −2.13). These results show that the visceral and subcutaneous adiposity volumes were smaller for diabetic patients, as opposed to participants who had diabetic-related conditions.

A contrast test for types of health conditions moderator effect was 2.46, which was positivly significant (*P* > 0.05, *Z* < 2.13). The positive value of the contrast test results suggests that differences between adipose tissue volumes were smaller in subcutaneous adiposity compared to visceral adiposity when the treatment group was composed of type 2 diabetes patients rather than prediabetic individuals (*P* < 0.05).

No indication of publication bias was detected via funnel plot analysis for either effect size estimate of visceral or subcutaneous adiposity.

## 5. Discussion

This research was the first study to use a quantitative meta-analysis to explore the distribution of the two primary types of abdominal adiposity, visceral and subcutaneous, in diabetic/prediabetic versus nondiabetic populations by systematically analyzing 41 relevant studies. Both visceral and subcutaneous adiposity volumes were significantly higher in diabetic/prediabetic groups, as opposed to nondiabetic group. The subcutaneous adiposity pooled effect size of 0.42 was relatively small, in comparison to the visceral adiposity pooled effect size of 0.69. This demonstrates that the differences in abdominal adiposity were higher for visceral adiposity than subcutaneous adiposity.

Gallagher et al. (2009) assessed total body adiposity, including visceral, subcutaneous, and intermuscular adipose tissue volumes via MRI and compared those adipose tissue distributions among healthy (*n* = 93) and type 2 diabetic (*n* = 93) subjects [80]. Diabetic patients possessed more total, visceral, subcutaneous, and intermuscular adipose tissues than the healthy subjects. The variations of the adipose distribution were greater for visceral adiposity than other types of adipose tissues, in both men and women [80]. However, diabetic populations had greater visceral and intermuscular adipose tissues and less subcutaneous adipose tissue than the healthy controls when the differences in covariates (ethnicity, gender, being diabetic or healthy, weight, height, age, and interaction of these factors) were adjusted [80]. Subsequently, being healthy versus diabetic was identified as one of the significant covariates suggesting that being diabetic is one of the critical factors in possessing higher amounts of visceral adiposity [80]. Similarly, significant disparities in abdominal adiposity distribution were identified between diabetic/prediabetic and nondiabetic participants in the current study.

The preponderance of evidence suggests that visceral adiposity has greater implications in terms of diabetes-related risk factors, as compared to subcutaneous adiposity [2, 12, 16, 17, 81–85]. Carr et al. (2004) demonstrated that both visceral fat and subcutaneous fat showed a strong association with metabolic syndrome criteria [84]. However, visceral fat exhibited the greatest relationship with these parameters, when controlling for insulin sensitivity and subcutaneous adiposity [84]. Similarly, Fox et al. (2007) reported a strong correlation between abdominal adiposity and metabolic risk factors, including blood pressure, fasting plasma glucose, triglycerides, and high-density lipoprotein cholesterol, as well as elevated odds of hypertension, impaired fasting glucose, and diabetes and metabolic syndrome in men and women (*P* < 0.05) [12]. Visceral adiposity was more closely linked to these factors, as opposed to subcutaneous adiposity [12].

In contrast, two older studies from 1990s found that subcutaneous adiposity is related more closely to diabetes in men, as opposed to visceral adiposity [86, 87]. One possible reason for this disparity might be that subcutaneous fat might differ according to its depth or location. Kelley et al. (2000) explored visceral adiposity and two different types of subcutaneous adiposity tissues (superficial and deep fat). Both visceral (*r* = −0.61, *P* < 0.001) and deep subcutaneous adiposity (*r* = −0.64, *P* < 0.001) exhibited a higher correlation with insulin resistance (measured by euglycemic clamp) than that of superficial subcutaneous (*r* = −0.20, *P* > 0.05) [88].

It is recognized that overaccumulated visceral fat tends to act as a malfunctional adiposity that induces excess storage of ectopic fat (muscle, epicardial, and liver fats) [17]. Consequently, abnormal free fatty acid metabolism may trigger dysfunctional release of adipokines [17]. Accumulation of those ectopic fats influences the metabolic profile and eventually increases the risks for developing metabolic syndrome [17], whereas subcutaneous fat has been recognized as a “healthy adiposity,” which may not adversely impact the development of metabolic syndrome [17].

Gender effects on central fat distribution are well known since men tend to deposit adipose tissue in the abdomen, whereas women are prone to the accumulation of adiposity in the gluteal-femoral area [20]. Moreover, men have an additional capacity to store an extended amount of visceral fat in the abdomen, as opposed to women [22, 89]. Yet, previous research has reported contradictory results that may have been influenced by the method of assessing central fat volume. In particular, a number of studies utilized a single slice of the umbilicus area such as the area between the 4th (L4) and 5th (L5) lumbar vertebra slices, in order to quantify adipose tissue in the abdomen. This single slice method may not always reflect overall abdominal fat mass. According to the CT measured L4-L5 slice by Kanda et al. (2007), men had a higher amount of visceral adiposity and a lower amount of subcutaneous adiposity than women, regardless of the presence of metabolic syndrome [43]. Similarly, men exhibited greater visceral fat and less subcutaneous fat than women, regardless of age [22, 89] and ethnicity [90]. Additionally, men had a higher percentage of visceral adiposity (13.3%) compared to the overall abdominal adiposity volume of women (6%) (*P* < 0.05) [22].

Ye et al. (2009) demonstrated that nondiabetic men exhibited a higher degree of visceral adiposity than nondiabetic women, whereas diabetic men had less visceral adiposity than diabetic women [40]. Also, the differences in visceral adiposity volumes between nondiabetic and diabetic individuals were higher in women, as opposed to men [40]. These results suggest that men are prone to possess a higher degree of visceral adiposity; yet, the increase in visceral adiposity is larger for women as they progress to diabetes. The present meta-analysis found that the differences in visceral adiposity volumes among diabetic/prediabetic versus nondiabetic groups were smaller for men, as compared to women (*P* < 0.05). It should be noted that no gender effect was detected for subcutaneous adiposity. These results support that diabetic women have more visceral adiposity in the abdomen area than do diabetic men. This significant gender moderator effect on visceral adiposity might be explained by the age of the women participants since a number of the subjects consisted of older women, who might be in their menopausal stage. Due to menopause-related changes in sex steroid hormones, postmenopausal women generally exhibit greater amounts of abdominal adiposity, especially visceral adiposity, compared to premenopausal women [91–94].

Previous studies that explored the efficacy of CT and MRI to measure abdominal adiposity volumes have been controversial. Several studies showed a consistent agreement of CT and MRI for measuring visceral and subcutaneous adiposity [95, 96], but others have reported a greater efficiency with MRI. Kullberg et al. (2009) and Tanaka et al. (2006) determined that visceral adiposity was underestimated when using MRI, as compared to CT (*P* < 0.05) [97, 98]. In addition, Seidell et al. (1990) demonstrated that no differences were found between CT and MRI measured subcutaneous adiposity volumes [99]. The current investigation observed that differences in visceral adiposity between diabetic/prediabetic versus nondiabetic groups were smaller when CT was used to measure the abdominal adiposity, whereas no moderator effect was identified for subcutaneous adiposity. These results suggest that CT may underestimate, or MRI may overestimate, visceral adiposity among diabetic/prediabetic and nondiabetic groups. Abdominal fat assessment of the CT or MRI umbilicus slices involves manual adjustment via customized software to quantify the abdominal adiposity tissue [34]. It is conceivable that this adjustment might have produced intra- and/or interobserver reproducibility errors. Additionally, the significantly smaller amount of visceral adiposity between diabetic/prediabetic versus nondiabetic groups while using CT compared to MRI may be explained by the error variances derived from different methods that are used to measure abdominal adiposity volumes. The specific location of the abdomen and the technique of single slice versus multiple slices may contribute to variations that influenced the results. It should be noted that most of the studies investigating the relationship between measurement of insulin resistance and visceral and subcutaneous fat used MRI measurements, thereby validating the measurement of visceral adiposity by MRI as a marker of insulin resistance.

Diabetic patients have more severe conditions than prediabetic individuals and central obesity is strongly associated with diabetes or diabetes-related symptoms. Thus, it is expected that those diagnosed with type 2 diabetes mellitus would possess a higher degree of abdominal adiposity compared to prediabetes [10]. The results regarding the type of health conditions for diabetic/prediabetic group moderator effect in the present study diverged from the expected outcome that diabetic participants would have a greater amount of abdominal adiposity than those with prediabetes. Rather, we observed that prediabetic participants had greater subcutaneous and visceral adiposity, as compared to diabetic patients.

It is possible that disease duration and certain medical treatments in diabetic patients affected abdominal adiposity. For example, therapeutic agents, such as thiazolidinedione, are known to affect fat distribution. However, limited information was available regarding the duration of diabetes and the type of medical treatment for the diabetic/prediabetic group so we could not explore these components as moderators. Only six studies provided the diabetes duration [33, 42, 52, 54, 55, 73] and four papers stated that the diabetic participants with current use of insulin or thiazolidinedione were excluded from the study [33, 56, 57, 63]. Furthermore, the results of this research may have been influenced by the different characteristics of the prediabetic group or other confounding factors. In addition, prediabetic groups were diverse in terms of degree of insulin resistance, metabolic syndrome, and impaired glucose tolerance. The majority of the diabetic group was Caucasians, whereas a number of the prediabetic groups varied in ethnicity, including African Americans, French, Indians, Japanese, and Koreans. Collectively, the above factors may have contributed to the disparities in visceral and subcutaneous adiposity.

A limitation of this study is that the causal relationship between diabetics and abdominal adiposity distribution was not clarified by the nature of the effect size, a standardized mean difference that was used in this research. Further meta-analysis that incorporates the relative risk or odd ratio effect sizes is needed to explicate the causal association between diabetes and abdominal adiposity distribution.

The inclusion of BMI as a moderator may have improved the accuracy of the results for accumulation of visceral and subcutaneous adiposity between diabetic/prediabetic and nondiabetic participants. But moderator analysis for BMI was not possible because most of the studies reported only the mean BMI values according to the groups and did not provide the numbers or percentages of the individual obese participants within diabetic/prediabetic and nondiabetic groups.

A second limitation is that most of the studies compared the quantity of abdominal adiposity volumes between diabetic groups and healthy controls. Whether or not the healthy controls were screened for prediabetes or undiagnosed diabetes is unknown. Therefore, the term “nondiabetic group” rather than “healthy controls” was used in this study to indicate the population who were nondiabetic or exhibited normal glucose tolerance.

This research focused primarily on comparison of the absolute quantity of abdominal adiposity between diabetic/prediabetic and nondiabetic groups. Future research could use the ratios of visceral adipose tissue to subcutaneous adipose tissue fat to explore the disparity of relative distribution of visceral subcutaneous adiposity between diabetic/prediabetic and nondiabetic population.

## 6. Conclusions

This paper explored variations in adipose tissue distribution among diabetic/prediabetic and nondiabetic populations by incorporating 101 effect sizes from 41 relevant studies via a comprehensive meta-analysis. The diabetic population possessed greater visceral adiposity than subcutaneous adiposity, and the differences were greater in visceral, as compared to subcutaneous, adiposity. Gender, method to assess abdominal adiposity volumes, and health conditions of being a diabetic/prediabetic were identified as crucial moderators that influenced the magnitude of abdominal adiposity distribution in these populations.

## Figures and Tables

**Table 1:** Characteristics and moderators of studies included in the meta-analysis investigating visceral adiposity distribution among diabetic/prediabetic versus nondiabetic populations.

First Author (reference)	Year	Type of health condition		Sample size (nondiabetic/diabetic)	Proportion of men, % (nondiabetic/diabetic)	Mean age, years (nondiabetic/diabetic)	Mean BMI, kg/m^2^ (nondiabetic/diabetic)	Adiposity assessment (method)	Characteristics of participants	Effect size (95% confidence interval)
		Diabetic/prediabetic	Nondiabetic							
Gaborit [33]	2012	Type 2 diabetes mellitus	Nondiabetes	17/13	—^a^	37/48	41.9/41.9	MRI^b^	Obese	4.72 (3.33, 6.12)

Baum [34]	2012	Type 2 diabetes mellitus	Nondiabetes	13/13	0/0	57/59	27.4/26.8	CT^c^	Postmenopausal women	0.44 (−0.34, 1.22)

Hoyer [35]	2011	Type 2 diabetes mellitus	Nondiabetes	386/103	51/54	51.2/57.9	23.8/25.3	CT	Japanese-American (Japanese ancestry)	0.73 (0.50, 0.95)

Hanley [36]	2011	Type 2 diabetes mellitus	Nondiabetes	1014/82	39/39	40.6/51.1	28.4/33.3	CT	Hispanic and African American	1.12 (0.89, 1.35)

Bozzetto [37]	2011	Type 2 diabetes mellitus	Nondiabetes	10/13	100/100	47/53	34.2/33.2	MRI	Obese men	0.30 (−0.53, 1.13)

Kim [38]	2010	Type 2 diabetes mellitus	Nondiabetes	21/38	100/100	28.6/29.2	24.8/26.8	CT	Young men	1.44 (−0.85, 2.03)

Goel [39]	2010	Nondiabetes with metabolic syndrome (impaired fasting glucose)	Nondiabetes without metabolic syndrome	65/35	60/37	29.1/36.1	21.5/27.5	MRI	Asian Indians	0.94 (0.51, 1.37)

Ye [40]	2009	Type 2 diabetes mellitus	Nondiabetes	86/28	100/100	53.6/57.6	24.2/26.9	MRI	Chinese men	0.57 (0.13, 1.00)

Ye [40]	2009	Type 2 diabetes mellitus	Nondiabetes	141/35	0/0	52.2/56.3	24.2/27.9	MRI	Chinese women	0.95 (0.56, 1.33)

Shin [41]	2009	Insulin resistance (impaired fasting glucose)	Noninsulin resistant	49/45	35/62	46/48.7	23.1/25.1	CT	Koreans	0.92 (0.50, 1.35)

Rijzewijk [42]	2008	Type 2 diabetes mellitus	Nondiabetes	28/38	100/100	54/57	26.9/28.1	MRI	Elderly	0.80 (0.30, 1.31)

Kanda [43]	2007	Type 2 diabetes mellitus	Without metabolic syndrome	147/111	100/100	63.6/58.1	21.3/27.3	CT	Japanese men	1.88 (1.59, 2.18)

Kanda [43]	2007	Type 2 diabetes mellitus	Without metabolic syndrome	94/67	0/0	61.2/60.4	22.6/27.2	CT	Japanese women	2.00 (1.62, 2.38)

Dekker [44]	2007	Type 2 diabetes mellitus	Nondiabetes	8/8	100/100	47.5/51	24.5/29.9	MRI	Middle-aged men	1.89 (0.71, 3.07)

Azuma [45]	2007	Type 2 diabetes mellitus	Nondiabetes	35/67	43/42	55/60	32.9/34.0	CT	Elderly	0.18 (−0.23, 0.59)

Suwa [46]	2006	Type 2 diabetes mellitus	Nondiabetes	7/24	0/0	47.6/51	24.1/26.1	CT	Japanese women	1.30 (0.39, 2.20)

Kuk [47]	2006	Metabolic syndrome (impaired fasting glucose)	Nonmetabolic syndrome	71/14	100/100	50.3/54.4	26.3/30.7	CT	Caucasian men	1.36 (0.75, 1.97)

Kanaya [48]	2006	Type 2 diabetes mellitus	Nondiabetes	2213/143	47/48	74/73	26.7/29.8	CT	Elderly Caucasian and African American	0.56 (0.39, 0.73)

Iwasaki [49]	2006	Type 2 diabetes mellitus	Nondiabetes	108/140	48/46	54.1/61.1	26.1/25.1	CT	Japanese	0.10 (−0.15, 0.35)

Virtanen [50]	2005	Type 2 diabetes mellitus	Nondiabetes	10/19	100/100	37/58	30.9/29.5	MRI	Obese men	−0.12 (−0.89, 0.64)

Virtanen [50]	2005	Type 2 diabetes mellitus	Nondiabetes	20/12	100/100	37/58	24.5/25.5	MRI	Nonobese men	0.53 (−0.20, 1.26)

Lee [51]	2005	Type 2 diabetes mellitus	Nondiabetes	10/9	100/100	47.5/51	24.5/29.9	CT	Middle-aged men	2.39 (1.21, 3.57)

Diamant [52]	2005	Type 2 diabetes mellitus	Nondiabetes	16/16	75/75	54/54	24.4/25.9	MRI	Men and postmenopausal women	1.04 (0.30, 1.78)

Kanaya [53]	2004	Type 2 diabetes mellitus with fasting plasma glucose ≥126 mg/dL	Nondiabetes (normal glucose tolerance)	298/298	100/100	73.5/73.7^d^	26.7/28.5	CT	Elderly Caucasian and African American men	0.48 (0.32, 0.64)

Kanaya [53]	2004	Type 2 diabetes mellitus with fasting plasma glucose ≥126 mg/dL	Nondiabetes (normal glucose tolerance)	221/221	0/0	73.5/73.7^d^	27.5/30.8	CT	Elderly Caucasian and African American women	0.97 (0.77, 1.16)

Gastaldelli [54]	2004	Type 2 diabetes mellitus with fasting plasma glucose <9 mM	Nondiabetes	8/7	—	49/53	31.6/34.3	MRI	Obese Caucasians and Mexican-American	0.72 (−0.33, 1.77)

Gastaldelli [54]	2004	Type 2 diabetes mellitus with fasting plasma glucose <9 mM	Nondiabetes	8/9	—	41/56	24.4/26.8	MRI	Nonobese Caucasians and Mexican-American	0.60 (−0.38, 1.57)

Gastaldelli [54]	2004	Type 2 diabetes mellitus with fasting plasma glucose >9 mM	Nondiabetes	21/14	—	49/54	31.6/33.9	MRI	Obese Caucasians and Mexican-American	2.15 (1.31, 2.99)

Gastaldelli [54]	2004	Type 2 diabetes mellitus with fasting plasma glucose >9 mM	Nondiabetes	21/14	—	41/52	24.4/26.5	MRI	Nonobese Caucasians and Mexican-American	1.99 (1.17, 2.81)

Anjana [55]	2004	Type 2 diabetes mellitus	Nondiabetes	82/82	46/46	45/45	24.0/26.1	CT	Asian Indians	0.43 (0.12, 0.74)

Ryan [56]	2003	Prediabetes (impaired glucose tolerance)	Nondiabetes	100/17	0/0	46.5/56.1	26.5/34.0	CT	Caucasian and African American women	1.25 (0.71, 1.79)

Kelley [57]	2003	Type 2 diabetes mellitus	Nondiabetes	15/83	—	45.7/51.5	33.7/34.0	CT	Obese	1.18 (0.61, 1.76)

Katsuki [58]	2003	Metabolically obese, normal weight individuals with normal glucose tolerance	Nondiabetes	20/20	95/95	33.1/34.3	21.0/23.5	CT	Japanese	3.18 (2.25, 4.11)

Hayashi [59]	2003	Impaired glucose tolerance	Normal glucose tolerance	71/57	52/54	39.9/39.1	23.2/23.7	CT	Japanese-American (3rd generation)	0.41 (0.06, 0.77)

Goodpaster [60]	2003	Type 2 diabetes mellitus	Normal glucose tolerance	815/397	100/100	73.7/73.9	26.5/28.1	CT	Elderly men	0.39 (0.27, 0.51)

Goodpaster [60]	2003	Type 2 diabetes mellitus	Normal glucose tolerance	823/329	0/0	73.4/73.5	26.7/30.0	CT	Elderly women	0.80 (0.67, 0.93)

Summers [61]	2002	Type 2 diabetes mellitus	Nondiabetes	6/6	50/50	55/56	24/29	MRT	Adults	0.73 (−0.44, 1.90)

Hernández-Ono [62]	2002	Type 2 diabetes mellitus	Nondiabetes	39/9	0/0	55.7/56.2	27.6/27.5	CT	Postmenopausal women	0.02 (−0.70, 0.75)

Liao [63]	2001	Impaired glucose tolerance	Normal glucose tolerance	307/77	51/45	49.4/60.3	23.6/24.3	CT	Japanese-American (Japanese ancestry, 2nd or 3rd generation)	0.44 (0.19, 0.70)

Liao [63]	2001	Type 2 diabetes mellitus	Normal glucose tolerance	307/212	51/45	55.1/60.3	24.5/24.3	CT	Japanese-American (Japanese ancestry, 2nd or 3rd generation)	0.50 (0.33, 0.68)

Boyko [64]	2000	Type 2 diabetes mellitus	Nondiabetes	211/65	50/62	61.1/63.3	24.1/24.9	CT	Japanese-American (2nd generation)	0.40 (0.12, 0.68)

Boyko [64]	2000	Type 2 diabetes mellitus	Nondiabetes	192/13	51/77	40.0/43.1	23.7/27.7	CT	Japanese-American (3rd generation)	1.64 (1.06, 2.23)

Nam [65]	1999	Type 2 diabetes mellitus	Nondiabetes	12/12	100/100	41.6/45.0	—	CT	Obese men	0.07 (−0.73, 0.88)

Mcneely [66]	1999	Type 2 diabetes mellitus	Nondiabetes	212/23	100/100	51.3/55.9	25.1/26.8	CT	Japanese-American men	0.70 (0.27, 1.14)

Mcneely [66]	1999	Type 2 diabetes mellitus	Nondiabetes	158/17	0/0	51.0/60.3	22.6/23.5	CT	Japanese-American women	0.45 (−0.06, 0.95)

Dvorak [67]	1999	Metabolically obese, normal weight	Nondiabetes	58/13	0/0	28/29	21.5/22.5	CT	Young normal weight women	0.62 (0.01, 1.23)

Vauhkonen [68]	1998	Type 2 diabetes mellitus with insulin sensitivity	Nondiabetes	5/5	100/100	40.1/41.3^d^	25.0/24.6^d^	CT	Men	0.49 (−0.77, 1.75)

Vauhkonen [68]	1998	Type 2 diabetes mellitus with insulin sensitivity	Nondiabetes	9/15	0/0	40.1/41.3^d^	25.0/24.6^d^	CT	Women	−0.36 (−1.19, 0.48)

Vauhkonen [68]	1998	Type 2 diabetes mellitus with insulin resistance	Nondiabetes	5/7	100/100	40.1/40.5^d^	25.0/28.8^d^	CT	Men	1.17 (−0.07, 2.40)

Vauhkonen [68]	1998	Type 2 diabetes mellitus with insulin resistance	Nondiabetes	9/11	0/0	40.1/40.5^d^	25.0/28.8^d^	CT	Women	0.09 (−0.80, 0.97)

Fujimoto [69]	1994	Impaired glucose tolerance	Normal glucose tolerance	93/22	100/100	39.6/40.2	24.5/26.8	CT	Japanese-American men (3rd generation)	0.85 (−0.37, 1.32)

Fujimoto [69]	1994	Impaired glucose tolerance	Normal glucose tolerance	79/36	0/0	40.8/39.8	22.5/23.3	CT	Japanese-American women (3rd generation)	0.65 (0.25, 1.05)

Lemieux [70]	1992	Altered insulin-glucose homeostasis	Normal insulin-glucose homeostasis	65/39	100/100	33.5/35.5	25.7/28.3	CT	French ancestry men	1.07 (0.65, 1.49)

Gray [71]	1991	Type 2 diabetes mellitus	Nondiabetes	12/24	0/0	37/57	35.4/38.6	MRI	Obese women	0.93 (0.21, 1.66)

^a^Data not available.

^b^Magnetic resonance imaging.

^c^Computed tomography.

^d^Data combined with men and women within the same study.

**Table 2:** Characteristics and moderators of studies included in the meta-analysis investigating subcutaneous adiposity distribution among diabetic/prediabetic versus nondiabetic populations.

First author (reference)	Year	Type of health condition		Sample size (nondiabetic/diabetic)	Proportion of men, % (nondiabetic/diabetic)	Mean age, years (nondiabetic/diabetic)	Mean BMI, kg/m^2^ (nondiabetic/diabetic)	Adiposity assessment (method)	Characteristics of participants	Effect size (95% confidence interval)
		Diabetic/prediabetic	Nondiabetic							
Karampinos [72]	2012	Type 2 diabetes mellitus	Nondiabetes	35/29	0/0	61.9/62.5	25.8/26.9	MRI^b^	Postmenopausal women	−1.10 (−1.62, −0.57)

Gaborit [33]	2012	Type 2 diabetes mellitus	Nondiabetes	17/13	—^a^	37/48	41.9/41.9	MRI	Obese	−1.29 (−2.08, −0.50)

Baum [34]	2012	Type 2 diabetes mellitus	Nondiabetes	13/13	0/0	57/59	27.4/26.8	CT^c^	Postmenopausal women	−0.04 (−0.81, 0.73)

Hoyer [35]	2011	Type 2 diabetes mellitus	Nondiabetes	386/103	51/54	51.2/57.9	23.8/25.3	CT	Japanese-American (Japanese ancestry)	0.26 (0.04, 0.48)

Hanley [36]	2011	Type 2 diabetes mellitus	Nondiabetes	1014/82	39/39	40.6, 51.1	28.4/33.3	CT	Hispanic and African American	0.54 (0.31, 0.77)

Bozzetto [37]	2011	Type 2 diabetes mellitus	Nondiabetes	10/13	100/100	47/53	34.2/33.2	MRI	Obese men	−0.82 (−1.68, 0.04)

Kim [38]	2010	Type 2 diabetes mellitus	Nondiabetes	21/38	100/100	28.6/29.2	24.8/26.8	CT	Young men	−0.15 (−0.68, 0.38)

Goel [39]	2010	Nondiabetes with metabolic syndrome (impaired fasting glucose)	Nondiabetes without metabolic syndrome	65/35	60/37	29.1/36.1	21.5/27.5	MRI	Asian Indians	1.54 (1.08, 2.00)

Shin [41]	2009	Insulin resistance (impaired fasting glucose)	Noninsulin resistant	49/45	35/62	46/48.7	23.1/25.1	CT	Koreans	0.21 (−0.19, 0.62)

Kanda [43]	2007	Type 2 diabetes mellitus	Nonmetabolic syndrome	147/111	100/100	63.6/58.1	21.3/27.3	CT	Japanese men	1.36 (1.09, 1.63)

Kanda [43]	2007	Type 2 diabetes mellitus	Nonmetabolic syndrome	94/67	0/0	61.2/60.4	22.6/27.2	CT	Japanese women	0.97 (0.64, 1.30)

Azuma [45]	2007	Type 2 diabetes mellitus	Nondiabetes	35/67	43/42	55/60	32.9/34.0	CT	Elderly	−0.05 (−0.46, 0.36)

Suwa [46]	2006	Type 2 diabetes mellitus	Nondiabetes	7/24	0/0	47.6/51	24.1/26.1	CT	Japanese women	0.07 (−0.77, 0.91)

Kuk [47]	2006	Metabolic syndrome (impaired fasting glucose)	Nonmetabolic syndrome	71/14	100/100	50.3/54.4	26.3/30.7	CT	Caucasian men	1.00 (0.41, 1.60)

Kanaya [48]	2006	Type 2 diabetes mellitus	Nondiabetes	2213/143	47/48	74/73	26.7/29.8	CT	Elderly Caucasian and African American	1.28 (1.11, 1.45)

Iwasaki [49]	2006	Type 2 diabetes mellitus	Nondiabetes	108/140	48/46	54.1/61.1	26.1/25.1	CT	Japanese	−0.14 (−0.39, 0.12)

Dubé [73]	2006	Type 2 diabetes mellitus	Nondiabetes	18/18	50/50	56.1/58.3	33.4/33.7	CT	Adults	−0.12 (−0.77, 0.53)

Virtanen [50]	2005	Type 2 diabetes mellitus	Nondiabetes	10/19	100/100	37/58	30.9/29.5	MRI	Obese men	−0.86 (−1.66, −0.07)

Virtanen [50]	2005	Type 2 diabetes mellitus	Nondiabetes	20/12	100/100	37/58	24.5/25.5	MRI	Nonobese men	0.17 (−0.55, 0.89)

Diamant [52]	2005	Type 2 diabetes mellitus	Nondiabetes	16/16	75/75	54/54	24.4/25.9	MRI	Men and postmenopausal women	0.51 (−0.19, 1.21)

Kanaya [53]	2004	Type 2 diabetes mellitus with fasting plasma glucose ≥126 mg/dL	Nondiabetes (normal glucose tolerance)	298/298	100/100	73.5/73.7^d^	26.7/28.5	CT	Elderly Caucasian and African American men	0.26 (0.10, 0.42)

Kanaya [53]	2004	Type 2 diabetes mellitus with fasting plasma glucose ≥126 mg/dL	Nondiabetes (normal glucose tolerance)	221/221	0/0	73.5/73.7^d^	27.5/30.8	CT	Elderly Caucasian and African American women	0.31 (0.13, 0.50)

Gastaldelli [54]	2004	Type 2 diabetes mellitus with fasting plasma glucose <9 mM	Nondiabetes	8/7	—	49/53	31.6/34.3	MRI	Obese Caucasians and Mexican-American	3.20 (1.67, 4.73)

Gastaldelli [54]	2004	Type 2 diabetes mellitus with fasting plasma glucose <9 mM	Nondiabetes	8/9	—	41/56	24.4/26.8	MRI	Nonobese Caucasians and Mexican-American	1.73 (0.61, 2.85)

Gastaldelli [54]	2004	Type 2 diabetes mellitus with fasting plasma glucose >9 mM	Nondiabetes	21/14	—	49/54	31.6/33.9	MRI	Obese Caucasians and Mexican-American	2.99 (2.20, 3.97)

Gastaldelli [54]	2004	Type 2 diabetes mellitus with fasting plasma glucose >9 mM	Nondiabetes	21/14	—	41/52	24.4/26.5	MRI	Nonobese Caucasians and Mexican-American	1.92 (1.10, 2.73)

Anjana [55]	2004	Type 2 diabetes mellitus	Nondiabetes	82/82	46/46	45/45	24.0/26.1	CT	Asian Indians	0.20 (−0.11, 0.50)

Ryan [56]	2003	Prediabetes (impaired glucose tolerance)	Nondiabetes	100/17	100/100	46.5/56.1	26.5/34.0	CT	Caucasian and African American women	0.87 (0.34, 1.39)

Kelley [57]	2003	Type 2 diabetes mellitus	Nondiabetes	15/83	—	45.7/51.5	33.7/34.0	CT	Obese	−0.50 (−0.05, 0.06)

Katsuki [58]	2003	Metabolically obese, normal weight individuals with normal glucose tolerance	Nondiabetes	20/20	95/95	33.1/34.3	21.0/23.5	CT	Japanese	1.09 (0.43, 1.76)

Hayashi [59]	2003	Impaired glucose tolerance	Normal glucose tolerance	71/57	52/54	39.9/39.1	23.2/23.7	CT	Japanese-American (3rd generation)	0.46 (0.11, 0.82)

Goodpaster [60]	2003	Type 2 diabetes mellitus	Normal glucose tolerance	815/397	100/100	73.7/73.9	26.5/28.1	CT	Elderly men	0.27 (0.15, 0.39)

Goodpaster [60]	2003	Type 2 diabetes mellitus	Normal glucose tolerance	823/329	0/0	73.4/73.5	26.7/30.0	CT	Elderly women	0.39 (0.26, 0.52)

Summers [61]	2002	Type 2 diabetes mellitus	Nondiabetes	6/6	50/50	55/56	24/29	MRI	Adults	−0.16 (−1.29, 0.98)

Hernández-Ono [62]	2002	Type 2 diabetes mellitus	Nondiabetes	39/9	0/0	55.7/56.2	27.6/27.5	CT	Postmenopausal women	0.23 (−0.50, 0.95)

Boyko [64]	2000	Type 2 diabetes mellitus	Nondiabetes	211/65	50/62	61.1/63.3	24.1/24.9	CT	Japanese-American (2nd generation)	−0.04 (−0.32, 0.24)

Boyko [64]	2000	Type 2 diabetes mellitus	Nondiabetes	192/13	51/77	40.0/43.1	23.7/27.7	CT	Japanese-American (3rd generation)	0.71 (0.14, 1.28)

Nam [65]	1999	Type 2 diabetes mellitus	Nondiabetes	12/12	100/100	41.6/45.0	—	CT	Obese men	−0.16 (−0.96, 0.64)

Dvorak [67]	1999	Metabolically obese, normal weight (impaired insulin sensitivity)	Nondiabetes	58/13	0/0	28/29	21.5/22.5	CT	Young normal weight women	0.70 (0.08, 1.31)

Vauhkonen [68]	1998	Type 2 diabetes mellitus with insulin sensitivity	Nondiabetes	5/5	100/100	40.1/41.3^d^	25.0/24.6^d^	CT	Adult men	0.46 (−0.80, 1.71)

Vauhkonen [68]	1998	Type 2 diabetes mellitus with insulin sensitivity	Nondiabetes	9/15	0/0	40.1/41.3^d^	25.0/24.6^d^	CT	Adult women	−0.17 (−1.00, 0.65)

Vauhkonen [68]	1998	Type 2 diabetes mellitus with insulin resistance	Nondiabetes	5/7	100/100	40.1/40.5^d^	25.0/28.8^d^	CT	Adult men	1.05 (−0.17, 2.27)

Vauhkonen [68]	1998	Type 2 diabetes mellitus with insulin resistance	Nondiabetes	9/11	0/0	40.1/40.5^d^	25.0/28.8^d^	CT	Adult women	0.61 (−0.29, 1.52)

Fujimoto [69]	1994	Impaired glucose tolerance	Normal glucose tolerance	93/22	100/100	39.6/40.2	24.5/26.8	CT	Japanese-American men (3rd generation)	0.67 (0.20, 1.14)

Fujimoto [69]	1994	Impaired glucose tolerance	Normal glucose tolerance	79/36	0/0	40.8/39.8	22.5/23.3	CT	Japanese-American women (3rd generation)	0.27 (−0.12, 0.67)

Lemieux [70]	1992	Altered insulin-glucose homeostasis (impaired glucose tolerance)	Normal insulin-glucose homeostasis	65/39	100/100	33.5/35.5	25.7/28.3	CT	French ancestry men	0.73 (0.32, 1.14)

Gray [71]	1991	Type 2 diabetes mellitus	Nondiabetes	12/24	0/0	37/57	35.4/38.6	MRI	Obese women	0.33 (−0.37, 1.03)

^a^Data not available.

^b^Magnetic resonance imaging.

^c^Computed tomography.

^d^Data combined with men and women within the same study.
